# The Temporomandibular Joint and the Human Body: A New Perspective on Cross Talk

**DOI:** 10.3390/dj12110357

**Published:** 2024-11-08

**Authors:** Marwa M. S. Abbass, Dina Rady, Sara El Moshy, Israa Ahmed Radwan, Al-Hassan Soliman Wadan, Christof E. Dörfer, Karim M. Fawzy El-Sayed

**Affiliations:** 1 Oral Biology Department, Faculty of Dentistry, Cairo University, Cairo 11435, Egypt; marwa.magdy@dentistry.cu.edu.eg (M.M.S.A.); dina.radi@dentistry.cu.edu.eg (D.R.); sarah.mahmoud@dentistry.cu.edu.eg (S.E.M.); esraa.ahmed@dentistry.cu.edu.eg (I.A.R.); 2 Stem Cells and Tissue Engineering Research Group, Faculty of Dentistry, Cairo University, Cairo 11435, Egypt; 3 Oral Biology Department, Faculty of Dentistry, Galala University, Attaka 15888, Egypt; amohamed6521@su.edu.eg; 4 Clinic for Conservative Dentistry and Periodontology, School of Dental Medicine, Christian Albrechts University, 43517 Kiel, Germany; doerfer@konspar.uni-kiel.de; 5 Oral Medicine and Periodontology Department, Faculty of Dentistry, Cairo University, Cairo 24105, Egypt

**Keywords:** temporomandibular joint disorders, TMD-related systemic diseases, therapeutic approaches, biomarkers, microvesicles, platelet-rich plasma

## Abstract

**Background:** As a unique joint that facilitates the articulation of the upper and lower jaws, the temporomandibular joint (TMJ) is concerned with several critical functions, such as speech and mastication. Pain that can become incapacitating is a result of temporomandibular disorders (TMDs), which are complex disorders affecting the masticatory muscles and the TMJ. Several anomalies and TMDs have an interdisciplinary relationship. Complementary and concurrent disorders may be caused by occlusal anomalies, psychological disorders, and changes in spine posture. **Methods:** This article examines the clinical characteristics of TMDs, their classification, their etiological factors, and the impact of TMJ disorders on the human body with reference to their anatomies and histological structures. **Results:** The clinical picture of some TMJ pathologies may be unknown, so certain biomarkers, such as cytokines, may be useful for an accurate diagnosis as they are frequently seen in TMJ disorders. Furthermore, novel therapeutic approaches that target pro-inflammatory cytokines and treat TMDs by using tissue engineering and regenerative medicine while permitting TMJ cartilage and bone regeneration may offer numerous benefits that require clinical translation. **Conclusions:** Implementation of recent modalities such as microvesicles and platelet-rich plasma in growth factors may provide a promising approach to enhance bone formation. In addition, we target different biological markers that give insights into the introduction of new pharmaceutical agents for therapy.

## 1. Introduction

One special synovial joint where both translational and rotational motions are possible is the temporomandibular joint (TMJ). It is also known as the ginglymoarthrodial type of joint because it has a hinge movement as well as a sliding movement between bony surfaces, like the diarthroidial joint [1].

Given that the TMJ is considered as one of the most frequently used joints in the human body, it is susceptible to wear and degeneration in the absence of adequate functional and occlusion relations [2]. 

## 2. Anatomy and Histological Structure of TMJ

The TMJ comprises the mandibular condyle, which articulates with the mandibular fossa [3]. The articular surfaces of the TMJ are unique, being encompassed by fibrous tissue instead of hyaline cartilage [3,4]. Anteriorly, the glenoid fossa is circumscribed by articular eminence [3]. Laterally, the fossa is limited by the root of the temporal bone’s zygomatic process, while medially it is limited by the sphenoid’s spine. Posteriorly, the mandibular fossa is limited by squamotympanic and petrotympanic fissures [3,4]. A fibrous, non-elastic capsule enveloping the joint is connected to the articular eminence anteriorly, the squamotympanic fissure posteriorly, and the margins of the glenoid fossa laterally. The capsule is connected to the condyle’s neck inferiorly [4]. 

An inward circumferential extension of the capsule forms an articular disc that divides the joint cavity into two synovial joint cavities [5] that harbour the synovial fluid. Lateral ligaments protect the TMJ from excessive movements [6,7], and collateral ligaments anchor the disc to the condyle [8]. The TMJ is also associated with muscles of mastication [9]. 

**Figure 1:**
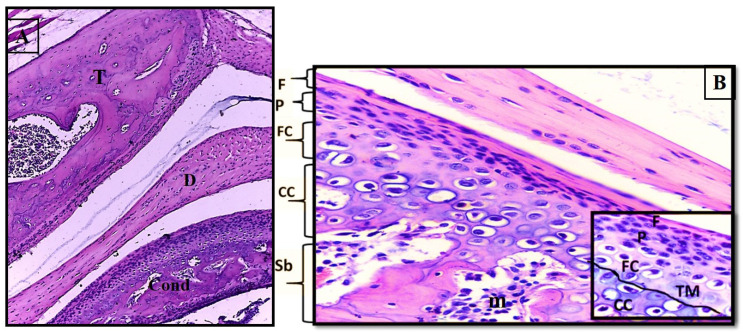
A histological picture of the TMJ of a normal rat showing (**A**) the mandibular condyle (Cond), biconcave articular disc (D), and temporal bone (T). (**B**) The condylar head has four zones: the fibrous zone (F), proliferative zone (P), fibrocartilaginous zone (FC), and calcified cartilage zone (CC) or hypertrophic zones and subchondral bone (Sb). The tidemark (TM) and bone marrow space (m) [10,18].

Histologically, the glenoid fossa is covered by a thin layer of fibrous tissue that thickens at the articular eminence’s slope [4]. Both the articular eminence and the condylar head are made of spongy bone [10]. There are four distinct layers covering the bony head of the adult condyle. The superficial articular surface is formed of collagenous fibrous tissue and some elastic fibres [11,12]. The proliferative layer is rich in collagen fibre types I and II with abundant undifferentiated mesenchymal cells, replacing adjacent layers in response to the functional demands. The fibrocartilaginous layer (the deep layer) is a fibrous layer with rounded cells that resemble cartilage-like cells. These cartilage cells are highly mature and are aligned parallel to the collagen fibres [13]. The deep zone is separated from the underlying calcified cartilage by a narrow, undulating line termed tidemark, as revealed in Figure 1, in which loadbearing areas appear wavy, while the non-loadbearing areas appear smooth [14]. The synovial membrane consists of a lining of synovial cells called synovial intima and an underlying connective tissue layer [15,16]. Using electron microscopy, two different cell types can be detected within the synovial intima, including macrophage-like type A cells in addition to fibroblast-like type B cells [5,17].

## 3. Temporomandibular Joint Disorders

The TMJ and masticatory muscles are affected by temporomandibular disorders (TMDs), which is also known as a temporomandibular dysfunction, a multifactorial condition that affects 5–12% of the population and causes pain that can become incapacitating [19]. The TMJ sounds and distorted mandibular movements accompanied by pain that interferes with everyday activities like eating and talking are among the signs and symptoms of TMDs [20,21,22,23,24]. Up to 60% of people, in general, of all ages and genders have experienced the symptoms and signs of TMDs at some point in their lives [25], and this percentage decrease to 15–37.5% in the adult population and 7% among adolescents [26,27,28].

**Figure 2:**
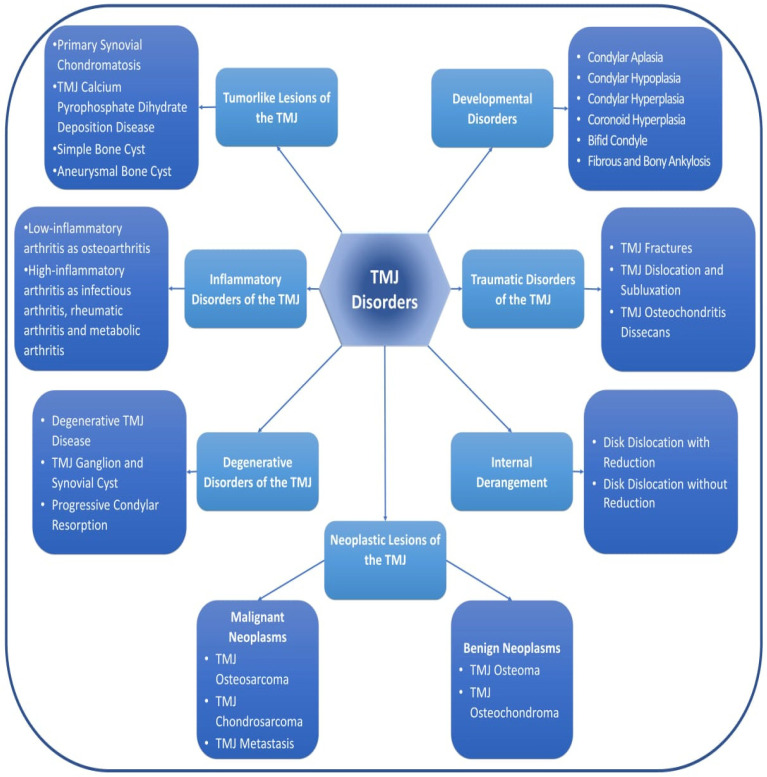
Classification of temporomandibular joint disorders (Tanaka et al., 2008; Salamon and Casselman, 2020) [30,31].

The Diagnostic Criteria for Temporomandibular Disorders (DC/TMD) have been the global standard for evaluating TMDs since 2014 [29]. The DC/TMD have two axes and their corresponding instruments: Axis I for physical diagnosis and Axis II for evaluating psychosocial state and impairment related to pain. The DC/TMD have been validated for several diagnoses using a standardized assessment methodology that includes a thorough investigation of the patient’s medical history and a clinical examination. An advanced diagnostic method that incorporates both patient history and clinical data allows for a high level of sensitivity and specificity in identifying certain subgroups of temporomandibular disorders (TMDs), resulting in high diagnostic accuracy for TMDs in adults [29]. According to Butts et al. [19], TMDs can be classified into three categories: TMDs related to muscle disorders, TMDs related to disc displacement with and without reduction, and TMDs related to joint pain. Recently, TMDs were classified based on their etiology into developmental disorders, traumatic disorders, inflammatory disorders, degenerative disorders, and neoplastic lesions of the TMJ as detailed in Figure 2 [30,31].

## 4. Etiological Factors Involved in TMDs

The etiologies of TMDs are multidisciplinary in nature [32] and comprise predisposing factors, initiating factors, and factors responsible for maintaining TMDs [32,33]. The predisposing factors that upregulate the susceptibility of TMD development include initiating factors with symptom onset, while the factors responsible for maintaining TMDs either curb the healing process or foster disease progression [34,35].

### 4.1. Age

The estimated percentages of TMD propagation among children and adolescents range from 6 to 68%. In a study carried out among adolescents with an age range from 12 to 18 years, 7% presented TMDs, clicks were recorded in 11%, mandibular fatigue and stiffness in 3%, and limitations in opening in 1% of the included subjects [36]. Controversially, in another study, joint sounds were experienced by 38% of the geriatric patients and muscle pain by 12%, while joint sounds were recorded only among 7% of young cases, with a significantly higher incidence of facial (7%), joint (16%), and muscle pain (25%) [37].

### 4.2. Gender Susceptibility and Estrogen Hormone

Previous epidemiological studies documented significantly higher frequency and severity of TMDs in females than in males. After puberty, TMDs mainly occur during the reproductive period [38], implying the possible role of estrogen and female hormones in the pathogenesis of TMD [39,40,41]. The presence of estrogen receptors in women’s TMJ cartilaginous tissue could be blamed for the higher TMD prevalence among women as compared to men [42].

The estrogen hormone was associated with altered joint metabolic functions and a significant increase in the laxity of joint ligaments in addition to its role in modulating the limbic system with an increased risk of painful stimuli. Rui-Yun et al. [43] revealed that 17-beta-estradiol can cause hypersensitivity of the inflamed TMJ in rats. It has been postulated that patients receiving replacement estrogen therapy experience a 30% increase in painful symptoms, while women using oral contraceptives experience a 20% increase [44]. Additionally, it has been demonstrated that women treated for TMDs while receiving combined oral contraceptives presented lower treatment outcomes than those who did not receive contraceptives [45]. Moreover, it has been authorized that relaxin and estrogen may cause TMJ disruption and activate matrix metalloproteinases (MMPs), which breakdown proteoglycans and cartilaginous collagen, thereby contributing to the breakdown of cartilage homeostasis [46]. Estrogen may exert its influence on the TMJ by impeding the proliferation of mandibular condylar chondrocytes through a pathway involving the estrogen receptor (ER)-β [47], through the conversion of estrone/17 β-estradiol to pro-inflammatory products in synoviocytes [48], or through the upregulation of Fas and caspase 3-related pro-apoptotic genes that worsened cartilage deterioration and subchondral bone destruction in a rat model of TMJ osteoarthritis induced by iodoacetate [49]. Surprisingly, estrogen may be a double-edged sword since the deficiency of estrogen led to serial degenerative changes in the TMJ, increased the cartilage thickness, and caused a reduction in the volume of the subchondral bone [50,51].

### 4.3. Trauma

There could be direct or indirect joint injuries. Ligament tearing can result from macrotraumas like direct blows to the face or microtraumas like bruxism, which causing an impulsive movement of the mandible [52]. Among pediatric patients, chin trauma causing unilateral and bilateral intracapsular or subcondylar fractures is blamed for the development of TMDs [53,54,55]. The literature is divided on whether acute trauma to the head or neck (such as whiplash in auto accidents) triggers chronic TMDs. Some authors considered these types of injuries as key factors in TMD development [53,56]. It has been reported that one of three subjects with whiplash trauma has a higher susceptibility of developing retarded TMD manifestations [57]. According to Davis [58], eating habits are compromised upon sustaining a neck injury. In a 400-patient study, TMDs were noted in 24.5% of the participants, and pain was positively connected with a history of trauma [59]. Klobas et al. [56] found that patients with whiplash injury had a higher incidence of TMDs with more severe TMJ symptoms than control patients (89% versus 18%).

On the contrary, Probert et al. [60] conducted a retrospective study comprising 20,673 car accidents victims in Australia where only 28 patients were diagnosed with TMDs. Accordingly, it was reported that whiplash injury could not be regarded as a triggering factor for the development of TMDs.

### 4.4. Occlusal Factors

Alterations in occlusion such as open bite, crossbite, excessive overbite, and overjet, occlusal interferences, midline discrepancies, and crowded and missing teeth have been recognized as TMD etiological factors [61,62,63], with a prevalence of 10–20% compared to other factors [64]. However, the specific mechanism by which malocclusion can induce TMJ pathology has not been clearly defined in the previous literature [65,66].

Rammelsberg [67] proposed an occlusal instability-based TMD developmental model resulting from a defective restorative procedure and the abrasion of posterior teeth. It has been revealed that patients with disc displacement experienced unilateral posterior crossbite, while patients with osteoarthrosis experienced excessive overjet, reduced overbite, and raised distance between centric relation and maximum intercuspation. Therefore, it was concluded that occlusal features might act as a TMD-inducing factor, and in another way, occlusal problems might occur secondary to TMDs [64].

The previous findings were disapproved by Hirsch et al. [68] who concluded that excessive overjet or overbite does not account for the development of joint sounds following the investigation of 3033 subjects. Additionally, Magnusson et al. [69] reported that occlusal features are weakly correlated to TMDs after the follow-up of 402 patients for 20 years. Koh et al. [70] also disproved in a meta-analysis that occlusal rebalancing can participate in the management or prevention of TMDs. A low incidence of malocclusion or improper occlusions in patients suffering from TMD signs or symptoms has been identified [71,72]. Further, a posterior crossbite could be blamed on an asymmetric muscle function; however, no certain association with TMDs has been identified [73,74]. The existence of mediotrusive involvements is considered a predisposing factor of disc displacement [75,76]. Concomitantly, an anterior open bite could be considered as an outcome of articular remodelling [77] instead of being the cause [78]. Moreover, the condylar position might act as a key player in TMD etiopathogenesis [79,80]. Padala et al. [81] and Weffort et al. [82] indicated that a significant number of dental inter-arch discrepancies and condylar displacements occur in patients with TMDs.

### 4.5. Parafunctions

Parafunctions could be defined as altered or impaired TMJ functions. Parafunctional habits such as bruxism, clenching, hyperextension, and other habitual behaviours might lead to TMDs as a result of joint overloading, which subsequently lead to synovial fluid alterations, cartilage breakdown, and other joint disorders [83].

#### 4.5.1. Chewing Gums

Miyake et al. [84] identified chewing gum and bruxism as risk factors for TMDs among 3557 students. Moreover, 323 females with an age range from 15 to 16 years, with an intense daily habit of chewing gum for more than 4 h, displayed a high prevalence of auricular pain and joint pain at rest and during various mandibular movements and joint noise and joint blockage during various movements of the jaw [85]. Karibe et al. [86] reported an increase in pain levels among both genders after chewing gum for six minutes, with a higher pain level observed among females, thus supporting higher female susceptibility.

#### 4.5.2. Bruxism

Children’s bruxism is typically impacted by psychological factors like high levels of stress or anxiety. The limbic system interprets stress as a stimulus that causes nervous tension. The masticatory organs receive this tension, which increases their hyperactivity and raises the muscle tone. The patient then tries, unconsciously, to grind their teeth and search for occlusal hooks [87,88,89]. The incidence of bruxism has been identified as 20% among adults, while a 38% propagation rate has been observed among children in whom only 5% presented TMD signs [83].

An association between bruxism and local blood flow disruption and ischemia has been previously reported. Local ischemia can have a depleting effect on the teeth, periodontium, masticatory muscles, and TMJ wellbeing in addition to pain due to ischemia [90]. Magnusson et al. [69] in a longitudinal study carried out over 20 years on 420 individuals reported a marked association between TMDs and bruxism. Huang et al. [91] also found a significant association between arthralgia, myofascial pain, and teeth clenching.

Bruxism is more commonly related to muscle dysfunction rather than joint dysfunction including disc displacement, articular cartilage degradation, and condylar bone remodelling [92,93,94].

### 4.6. Joint Hyperlaxity and Joint Hypermobility

Regarding the relationship between TMDs and systemic joint hypermobility, some authors have reported that no correlation exists, while others have deduced a direct relationship between both of them. Kavuncu et al. [95] reported a more frequent incidence of TMDs in patients with local and general hypermobility than controls.

These findings are consistent with a previous study conducted by De Coster et al. [96]. Thirty-one subjects with the Ehler–Danlos syndrome, a genetic disorder characterized by excessive joint flexibility, revealed TMD signs and symptoms with chronic dislocations. Gazit et al. [97] reported recurrent dislocations, chronic pain, and subluxations in the TMJ. The authors highlighted that most of the patients affected by the Ehler–Danlos syndrome showed signs of neck-related chronic headaches and jaw- or TMD-related facial pain.

The correlation between TMDs and generalized joint hypermobility has also been investigated [98,99]. The correlation has been emphasized by the fact that 71.4% of patients with generalized joint hypermobility had symptoms of TMDs including displacement without reduction (85.7%), myofascial pain (69%), and TMJ pain (61.9%) [100]. On the contrary, Conti et al. [101] found no association between systemic hyperlaxity and TMDs.

### 4.7. Posture

The neuromuscular and skeletal system’s method of maintaining balance in response to gravity is referred to as posture [102]. The human body naturally stabilizes damaged areas by facilitating greater movement in other parts [2]. The stomatognathic system and the craniovertebral joints actively participate in maintaining the correct position of the skull together with the TMJ in relation to the body; their movements are performed in a coordinated fashion, subsequently affecting the pattern of walking and standing [103].

Accordingly, alterations in the body posture involving the cervical region could induce TMDs owing to their effect on the condylar position and by supporting muscle tension [104,105]. It is common for patients with TMDs to present anteriorly positioned heads [106,107]. Alcantara et al. [108] in their review concluded that resolving cervico-cranio-mandibular dysfunction and diagnosing and treating spinal and extra-spinal subluxations may assist infants with breastfeeding difficulties. Cervical disturbances are correlated with bruxism, TMJ pain or limitation, mobility, or TMJ sound according to Hozl et al. [109].

Data from studies revealed that an alteration in chewing could cause a new mandibular position, therefore establishing a new posture [110,111,112]. Moreover, a review that considered 11 studies reported a correlation between TMJ anomalies and the vertebral column’s dysfunctions. Reciprocally, a number of studies have revealed no correlation between pain in the TMJ and occluded-postural anomaly [113,114,115,116,117].

### 4.8. Orthodontic Treatment

As per the pervious literature, no direct relation can be detected between orthodontic treatment and TMDs, regardless of any premolar extraction prior to treatment [118]. Despite the fact that the position of the teeth dictates occlusal stability and vertical dimension of the face [119], Kim et al. [120] found only 1 out of 31 reviewed articles that revealed a correlation between orthodontic treatment and the prevalence of TMDs. A similar finding was reported by Mohlin et al. [121] following a study on 337 patients in Sweden.

### 4.9. Psychological Factors

The pathogenesis of TMDs may be predisposed by psychological variables such as emotional behaviour, stress, and personality disorders [122,123,124]. Stress causes hyperactivity and exhaustion in the muscles, consequently resulting in muscular spasms, disharmony of occlusion, joint disturbances, and degenerative arthritis. It has been suggested that patients suffering from myofascial pain with underlying osteoarthritis, arthralgia, or arthritis are more likely to show more advanced stages of depression compared to those with disc displacement [125,126,127,128,129,130,131,132]. Manfredini et al. [133] carried out a study using stress measurement questionnaires and disclosed that stress was significantly higher in patients with TMDs. In a study utilizing stress measurement questionnaires, Madani et al. [134] discovered that stress was a major factor in the genesis of TMDs and concluded that risk factors like early contact, clenching and grinding, and joint injuries were not as significant as stress in the development of TMDs.

Surprisingly, pain and other TMD symptoms are blamed for either causing or exacerbating the development of psychic diseases and depression [127,128,130,132].

### 4.10. Hereditary and Genetic Factors

The genetic predisposition hypothesis of TMDs needs to be further verified. Even though several genes were suggested to be associated with TMDs [135], Michalowicz et al. [136] reported a lack of genetic factors’ relevant effect upon TMD occurrence among a group of 494 monozygotic and dizygotic twins. Additionally, no single-nucleotide polymorphisms could be linked to the occurrence of TMJ osteoarthritis [137].

## 5. Cross Talks Between TMJ Disorders and the Human Body

The temporomandibular joint, the only joint in the skull, may indicate the presence of systemic diseases, or it could be the repercussion. In a questionnaire-based study, the correlation between TMDs and systemic diseases was investigated. The patients surgically treated for painful clicking or chronic closed lock presented a higher probability of suffering from pneumonia, asthma, allergies, headaches, hypermobility, previous orthodontic treatment, and orofacial trauma than the control group [138].

### 5.1. TMDs and Nervous System Involvement

The TMJ is well innervated by the branches of the trigeminal nerve. TMDs have the potential to have systemic effects on the central nervous system as well as cause neural inflammation in the peripheral nervous system near the site of injury. The consequences of neural inflammation include activated microglia and increased cytokine expression [139].

A study comparing patients with comorbid TMDs and systemic/neurologic conditions before and after using a custom-made dental orthotic revealed a marked elevation in biomarkers for inflammation (IL-1β, TNF-α, IL-6, IL-17A), pain (SP, CGRP), and tissue-destruction enzymes (PAD-4) in their synovial and salivary fluids. Additionally, the functional magnetic resonance imaging activity of the brain indicated a decreased blood flow to the anterior frontal lobes, especially in patients who were not wearing their customized dental orthotics [139]. Moreover, the TMJ myofunctional disturbances due to trauma and repetitive loading change the microenvironment of the joints and the innervated constitutive tissues’ biomechanics. Changes in the joint microenvironment have the potential to activate peripheral pain sensors, which then transmit pain signals to the brain and spinal cord for processing and perception leading to chronic pain [140].

### 5.2. TMDs and Spinal Pain

Using the national data from Korea, the relationship between spinal pain and TMDs was studied. The incidence of spinal pain among patients with TMDs was estimated as 48%, whereas among the control group, it was 34%. The severity of TMDs was positively correlated with higher spinal pain [141]. These data assumed that treating the TMJ injury reduces neural inflammation and alleviates spinal pain.

### 5.3. TMDs Correlated to Otolaryngologic and Ophthalmologic Disorders and Chronic Diseases

Logistic regression analysis carried out among 17,575 Korean subjects revealed that participants who suffered from asthma, migraine, osteoarthritis, thyroid dysfunction, depressive symptoms, tinnitus, hearing difficulties, dizziness, rhinitis, and xerophthalmia had higher TMD prevalence as compared to individuals without such diseases [142]. Moreover, Assouan et al. [143] reported that TMJ involvement and pre-auricular swelling were predominant signs in extra-pulmonary and extra-spinal tuberculosis. These findings suggest the need for multitarget therapy to effectively address this phenomenon.

About 85% of patients with TMDs may experience aural symptoms like vertigo, tinnitus, otalgia, and dizziness. Given that the Meckel’s cartilage participates in the early development of both the middle ear and TMJ, the occurrence of the auditory symptoms may be explained. Additionally, the ear and the masticatory muscle share innervation [144,145].

### 5.4. Correlation Between Fibromyalgia and TMDs

The TMJ alterations are the crucial first step in an early fibromyalgia diagnosis [28,146,147,148]. Upon reviewing articles correlating fibromyalgia and TMDs, it was deduced that both diseases do not present concomitantly, yet fibromyalgia may induce the pathogenesis of TMDs. Fibromyalgia is characterized by diffuse pain that compromises the nociceptive pathways, resulting in greater sensitization to muscles’ pain, damages the nervous system, and leads to TMD development [149].

### 5.5. TMDs and Headache

Headache is the most common neurological disease. The comorbidity of headaches and TMDs has been emphasized, with high prevalence rates in different studies where 22%–67.5%–82.8% of patients with TMDs were reported to suffer from headaches [150,151]. It is possible to identify central sensitization mechanisms and shared neural pathways as the primary causes of the correlation between TMDs and primary headaches [152].

#### 5.5.1. Tension Headache

The most prevalent primary headache affecting the general public is the tension-type headache [153]. According to research conducted by Franco et al. [154], up to 30.2% of patients with TMDs may experience tension headaches. Emshoff et al. [155] reported that tension headaches were experienced by half of the patients with TMDs in their study.

#### 5.5.2. Migraine

Migraine is a chronic headache that is typically one-sided, pulsating, and varying in intensity [156]. TMDs and migraines are comorbid diseases [157], and a high propagation rate of TMDs among patients with migraine was estimated as 56.1% [158]. Costa et al. [152] pointed out that migraine increases TMJ pain and worsens TMD symptoms. Gonçalves et al. [159] deduced in their study that, if migraine is accompanied with TMDs, migraine pain could be alleviated if both conditions are treated in synchrony.

### 5.6. TMDs and Systemic Lupus Erythematosus

In a study conducted to investigate the incidence of oral manifestations in association with TMDs, 95.8% of patients with systemic lupus erythematosus revealed both oral and TMJ symptoms. In particular, limited protrusion was prevalent among patients with lupus (85.2%) versus (5.4%) controls. A total of 59.3% of patients with lupus also displayed a limited left lateral movement. In addition, bruxism and tongue indentations were significant in the lupus group (72.7%) as compared to control subjects [160].

### 5.7. TMDs and Levels of Salivary and Plasma Pain Markers

In a group of patients suffering from chronic TMD myalgia, the concentrations of serotonin, glutamate, brain-derived neurotrophic factor (BDNF), nerve growth factor (NGF), and substance P in saliva and plasma were analyzed utilizing colorimetric assay, ELISA, and multiplex electro-chem-iluminescence assay panel. Patients with TMDs expressed higher salivary and plasma levels of glutamate than controls, highlighting their key role in the TMD myalgia pathophysiology, with no correlation with pain levels. Salivary BDNF and NGF were lower in patients compared to control subjects; on the contrary, plasma BDNF was higher in patients than controls. NGF and BDNF concentrations were reported to be correlated to the levels of psychological distress [161].

### 5.8. TMDs and Rheumatic Diseases

Rheumatic diseases are a group of diseases with autoimmune and inflammatory bases [162]. In a study carried out by Iordache et al. [163], it was demonstrated that the involvement of the TMJ is higher among all rheumatic disease groups regardless of the nature of the disease, i.e., inflammatory or degenerative. In an attempt to investigate rheumatic diseases as risk factors for TMDs, a total 143 patients were investigated using bone scintigraphy. The findings showed that rheumatoid arthritis, axial spondyloarthritis, and peripheral spondyloarthritis can all function as TMD predisposing factors by inflaming the TMJ. Moreover, C-reactive protein and erythrocyte sedimentation rate can be used as markers of advancing TMJ inflammation [164].

#### 5.8.1. Rheumatoid Arthritis (RA)

RA is a severe autoimmune disease that damages joints and impairs their ability to function [165]. Rheumatoid factor (RF) and anti-cyclic citrullinated (anti-CC) antibodies are widely used as markers for RA diagnosis. However, RF can also be detected in various inflammatory conditions, including RA [166,167]. Antinuclear antibodies (ANAs) can also be detected in RA [168]. Diagnosing RA should include the careful evaluation of both clinical symptoms and titers [166]. It is estimated that between 0.5 and 1.0% of adults in industrialized nations suffer from RA [169]. The joint structure may still be damaged in individuals without any clinical symptoms of RA involving the TMJ; this damage is only detectable through imaging techniques [170,171]. Pain in the craniofacial area, as well as pain in the joints, muscles, or both, is present in 75% of patients with RA with TMJ disorders [170]. Later stages may include muscle spasms, jaw movement restriction, and joint stiffness, which may occur in 66% of patients [172,173]. The abnormal position of the articular disc, abnormal articular disc morphology, or articular exudate are detected by magnetic resonance imaging in 95.2%, 83.3%, and 30.9% of cases, respectively [174]. Patients with TMDs have alterations in the shape of the disc, and patients with RA have further resorption in the condylar bone, disrupting the relationship between both [175]. Axial skeleton involvement was observed in severe and long-standing cases of RA [176,177]. Typically, TMJ involvement is discovered following disease progression [178,179]; therefore, it can be assumed that the duration of axial joint involvement in patients with rheumatoid arthritis correlates with TMJ involvement [164].

#### 5.8.2. Osteoarthritis (OA)

**Figure 3:**
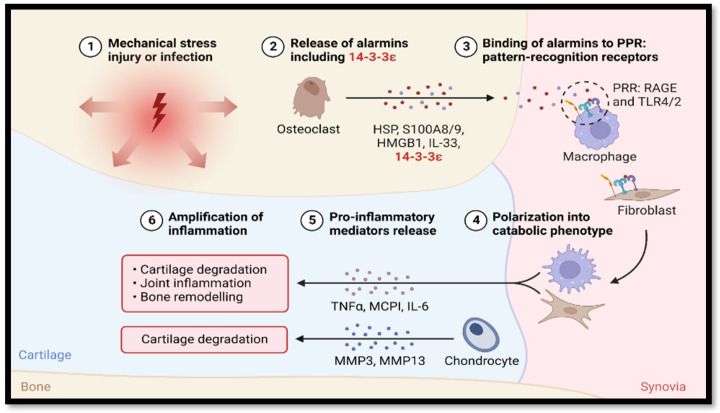
Pathogenesis of osteoarthritis (OA) disease.

**Figure 4:**
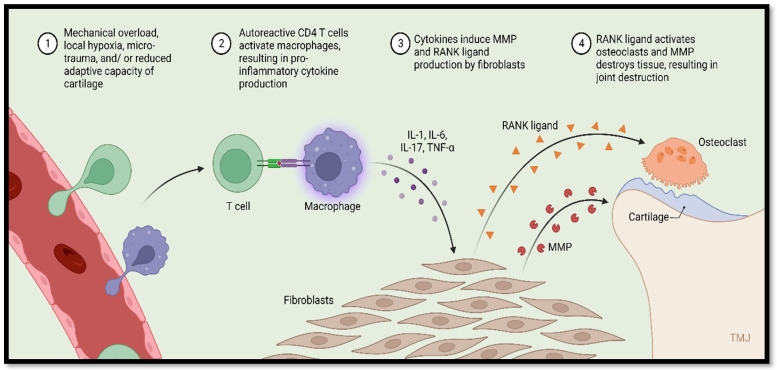
Pathogenesis of temporomandibular joint osteoarthritis (TMJ-OA).

The most prevalent inflammatory condition affecting the joints is OA. Microinjuries and biological factors play a role in its progression (Figure 3). Chronic inflammation in the synovial tissue, subchondral bone remodelling, and cartilage degradation are the hallmarks of OA in the TMJ (Figure 4). Clinical symptoms may include muscle pain and impairment of jaw movements [40,180,181,182,183,184]. Typical erosion, flattening of the condylar bone and articular eminence, osteophytes, articular cysts, and loss of the joint space were recorded in patients with TMJ-OA [182,185]. A study carried out by Kothari et al. [186] revealed that patients with arthritic pain in the TMJ were less sensitive to cold, warm, and tactile sensations than patients with osteoarthritis. It is noteworthy that investigating the biological markers is necessary since a weak correlation exists between the occurrence of bone lesions and the clinical symptoms of TMJ-OA [185,187].

#### 5.8.3. Juvenile Idiopathic Arthritis (JIA)

JIA is the predominant condition in the field of pediatric rheumatology, with the TMJ being affected in around 80–87% of patients [188,189]. Arthritis often begins before the age of 16 and the symptoms must persist for a minimum of six weeks. However, it can either reoccur or manifest for the first time in an adult [162,190]. TMJ symptoms caused by JIA may manifest as face pain, headaches, malocclusion, difficulty chewing, or a mandible that is positioned further back than normal (retrognathic mandible) [191]. In a study conducted by Kirkhus et al. [192], it was found 63% of patients with JIA who had TMJ involvement developed disc abnormalities, such as a flat disc or the fragmentation of the disc. Munir et al. [193] reported a 75% and 62.5% prevalence of TMJ effusion and synovial thickening among patients with JIA, which was detected using magnetic resonance imaging. Conversely, a literature review investigating the clinical variables of TMJ synovitis in JIA did not confirm the etiopathalogical correlation. This could be because of the inadequate quantity of evidence and heterogeneity of the studies conducted [194].

### 5.9. Occlusal Changes Secondary to Temporomandibular Disorders

The development of malocclusion in association with symptoms and signs of TMDs could be a complaint of a group of patients [182]. Thus, occlusal alterations may indicate the existence of TMDs that, when controlled, a normal occlusion could be re-established [195]. The most frequently occurring occlusal abnormalities resulting from TMDs are described below.

#### 5.9.1. Anterior Open Bite

An anterior open bite is widely presented in patients with TMJ degenerative diseases. TMJ-OA with functional overloading might result in a collapse of joint tissues. Condylar resorption occurs when it is present in both TMJs, resulting in a breakdown of their structure and a reduction in the height of the ramus. This leads to a slow backward movement of the lower jaw, causing a condition known as anterior open bite [196]. Additionally, individuals with RA suffer from more occlusal interferences, a bigger difference between centric relation and maximum intercuspation, and a reduced vertical overbite [197,198]. Contrary to the minor open bite induced by TMJ-OA, the aggressive and severe open bite could be induced in a relatively short time due to idiopathic condylar resorption [197]. This disorder predominantly affects females and is impacted by hormonal fluctuations, as well as external influences such as orthognathic surgery or other traumatic events [198].

#### 5.9.2. Unilateral Posterior Open Bite

Unilateral condylar resorption occasionally pushes the cervical condyle inward, causing the mandible to shift towards the affected side. An anterior open bite is consequently accompanied by a posterior open bite on the opposing side [199]. Due to the dense innervation and vascularization of TMJ retrodiscal tissues, they are more prone to experiencing an inflammatory cascade that can result in joint effusion caused by the deposition of inflammatory fluids that impede the condyle’s proper fitting into the glenoid fossa. The outcome is the development of a posterior open bite on the same side, which is accompanied by a significant contact with the canine region on the opposite side [200].

## 6. Clinical Features of TMDs

The presentation of clinical features of TMDs may vary. Pain is the primary manifestation of the majority of TMDs and the primary impetus for patients to pursue treatment. The pain may be caused by the contraction of masticatory muscles, which stimulates the extravascular secretion of inflammatory cytokines throughout the TMJ [201]. Approximately 13.5% of patients may exhibit a clicking sound when they are swallowing, opening, or closing their mouth. This sound may indicate articular disc disorders of the TMJ [202]. Additionally, restricted mandible movement may be an indication of TMDs. During the jaw movement, symptoms such as joint locking, tenderness in the jaw muscles, and deviation or deflection of the mandible may be observed. Degenerative joint diseases can arise from secondary inflammation of the synovial membrane. This inflammation triggers a series of events that terminate in fibrosis and muscle weakness, ultimately leading to the degradation of the articular surfaces and the failure of the lubrication system [203]. Headaches and aural symptoms are common among patients with TMDs, as discussed earlier.

Diagnosis of TMDs might be challenging due to TMD symptoms’ heterogeneity, which affects the proper planning of treatment [204]. TMD diagnosis relies on history and physical examination that are dependent on the human factor and is therefore liable to some symptom misinterpretations, leading to the misdiagnosis of TMDs [205].

This highlights the importance of the research diagnostic criteria for TMDs (RDC/TMD) that have been widely used as a diagnostic protocol for TMD research together with listening properly to patient feedback [206]. Hence, new approaches are crucial for specific TMD diagnoses by exploring more sensitive diagnostic biomarkers involved in TMDs [207].

## 7. Biomarkers for TMDs

**Figure 5:**
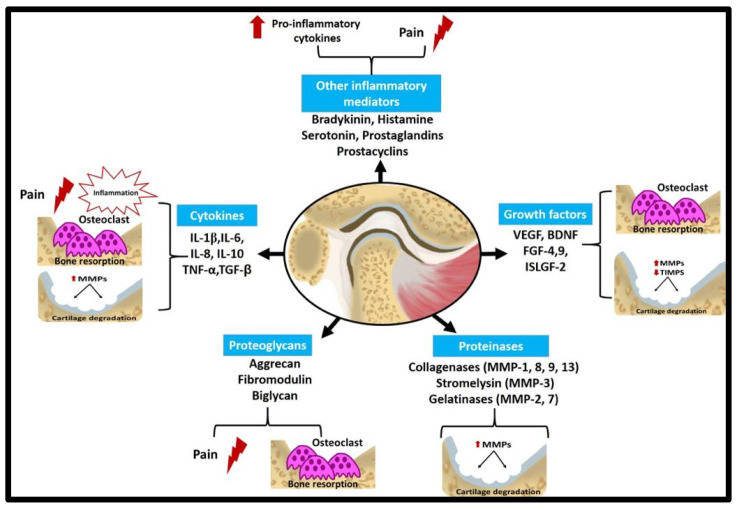
Biological markers involved in temporomandibular disorder pathogenesis.

A biomarker can be defined as a specific characteristic or marker that can be measured objectively to give an indication of a specific normal biological or pathogenic processes or indicate a biological response to a given pharmacological intervention [208]. Biomarkers present a highly sensitive and a specific diagnostic tool that are detected prior to the clinical symptoms and are reversible after proper treatment [209]. However, up to date, there are no biomarkers for TMDs in clinical use. Synovial fluid, saliva, and blood can be potentially contain the early diagnostic markers for TMDs [205,210]; see Figure 5.

### 7.1. Cytokines

Cytokines, which are diminutive proteins, function as the mediators of inflammation in complex immunological networks [211]. Cytokines include interleukins (ILs), tumour necrosis factor (TNF), interferons, chemokines, and lymphokines. IL-1, IL-6, IL-8, IL-12, and TNF are pro-inflammatory, while IL-4, IL-10, and transforming growth factor-beta (TGF-ꞵ) are anti-inflammatory [211,212]. IL-6 and TGF-ꞵ can have both pro-inflammatory and anti-inflammatory effects [212]. However, there is an ongoing debate over the results concerning cytokines, as TMDs such as OA and internal derangement are associated with high levels of pro-inflammatory cytokines [213]. These mediators release proteinases and other inflammatory molecules that contribute to cartilage and bone degradation.

The elevated levels of TNF and IL-1ꞵ were among the first cytokines detected in the synovial fluid of patients with internal TMJ derangement with and without degenerative changes compared to healthy controls [214,215]. Moreover, the high levels of IL-6 and IL-8 in the synovial fluid of patients with internal derangement and OA indicated their possible role in TMDs [216]. IL-6 concentration in the synovial fluid showed a positive correlation with synovitis [217]. IL-1β, TNF-α, and IL-6 were highly expressed in albino rats’ TMJ during induced osteoporosis [10]. IL-6 is responsible for the differentiation of osteoclasts and bone resorption by enhancing the interaction between osteoclasts and osteoblasts, serving as a potential hypothesis for OA pathogenesis [218].

Further analysis of blood samples revealed that TGF-β1, IL-8, IL-1 receptor antagonist (IL-1ra), and monocyte chemotactic protein (MCP-1) could serve as therapeutic targets and diagnostic indicators for pain management in patients with TMDs [219]. IL-1ꞵ stimulated the upregulation of MCP-1 in TMJ synoviocytes, which in turn attracted monocytes to the inflammatory synovial tissue [220]. Furthermore, IL-1ꞵ upregulated hyaluronic acid synthase 3 in synovial tissues during inflammation [221] and human TMJ disc tissue in patients afflicted with internal derangement [222]. TGF-ꞵ augmented hyaluronic acid synthase 1 and 2 production [223].

TNF-α contributes to the pathogenesis of synovitis in addition to TMJ bone and cartilage degeneration as its level in the synovial fluid of patients with internal derangement was increased in correlation with the disease stage [224]. Additionally, a significant positive correlation was observed between the level of TNF-α in the synovial fluid and the presence of TMJ pain during maximal jaw opening in patients diagnosed with chronic inflammatory connective tissue disease [225]. The decreased TNF-α level in the synovial fluid after treatment with glucocorticoids in patients with chronic inflammatory TMDs was associated with pain elimination upon maximum jaw opening [226]. TNF’s modulatory effects on tissue degradation and pain in TMDs are directly through its interaction with TNF receptors or indirectly through the induction of other pro-inflammatory cytokines (IL-1, IL-6, and IL-8) and prostaglandin, which supports inflammation by suppressing T helper 1 and natural killer cells as well as regulating effector T cells versus regulatory T cells [226,227,228]. Recently, IL-1, IL-6, and TNF-α were referred to as OA markers, and with moderate physical activity, their expression in the synovium of an OA-induced rat model was reduced [229]. The levels of IL-10 were elevated in healthy TMJs compared to the ones with disc derangement [230], indicating that the lack of IL-10 may contribute to the development of OA.

### 7.2. Other Inflammatory Mediators

Bradykinin is a vasodilator that mediates inflammation, and as bradykinin is associated with increased tissue perfusion, it could be involved in TMDs [231]. The role of bradykinin could be attributed to its interaction with specific receptors present on inflammatory cells, promoting the synthesis of IL-1 and the TNF. The synovial fluid levels of bradykinin in patients suffering from internal derangement and OA showed positive correlation to the degree of synovitis; therefore, it might be beneficial to index the degree of synovitis [232].

Histamine is another inflammatory mediator contributing to TMJ inflammation when released from degranulated mast cells due to tissue aggression [233,234]. The histamine level was higher in TMJ-OA than in other TMDs, with a positive correlation between pain and its concentration [235]. Histamine induces nociception indirectly through stimulating serotonin release [236]. Additionally, serotonin was significantly increased in patients with OA associated with pain and mandibular movement reduction [237]. Serotonin mediates pain through the activation of β1 and β2 adrenoreceptors in the TMJ in addition to the release of prostaglandins [238].

Prostaglandins and prostacyclins are produced from arachidonic acid by the cyclooxygenase enzymes during inflammation [239]. Both act as modulators in the late periods of inflammation, thereby augmenting histamine, serotonin, and bradykinin levels. This action is mediated through increasing their receptors’ sensitivity, resulting in increased local pain [234]. Prostaglandin E2’s high concentration in the synovial fluid that is related to TMJ allodynia highlights its role in developing and maintaining inflammation [240]. When all of the evidence is assessed together, TMDs are associated with a significant upregulation in the levels of prostaglandins and other inflammatory markers.

### 7.3. Proteinases

Matrix metalloproteinases (MMPs) are a group of proteases released during inflammation stimulated by IL-1ꞵ. Many MMPs were involved in TMDs, where high levels of collagenases (MMP-1, MMP-8, MMP-9, MMP-13), stromelysin (MMP-3), and gelatinases (MMP-2, MMP-7) were identified in the TMJ synovial fluid and joint’s synovial membrane as well as in the disc and condylar cartilage in cases of OA or joint’s internal derangement [214,241,242,243]. The high levels of MMP-2, MMP-8, and MMP-9 in the synovial fluid of patients with mild internal derangement were interpreted as reflections of the active phase of TMJ destruction [242]. Moreover, MMP-7 and MMP-9 were overexpressed in patients’ synovial tissue with anterior disc displacement without reduction [244].

Synovial macrophages, fibroblasts, and chondrocytes were claimed to release MMPs that destroy the TMJ cartilage in OA [245,246]. Specifically, MMP-1, MMP-3, and MMP-9 were proven to be synthesized in human TMJ synovial cells in vitro [247,248]. Fibroblast-like type B cells are mainly responsible for the secretion of collagen type I and II, fibronectin, and glycosaminoglycans into synovial fluids [5,249,250,251]. The overexpression of MMP-7 and MMP-9 in patients with severe TMJ dysfunction was attributed to fibroblast-like type B secretory activity [244]. Despite the ongoing research, there is a poor understanding of the exact mechanism to target specific MMPs [252]. Discovering a pharmaceutical agent that could target MMPs involved in TMDs could provide an advanced route for treating TMDs.

### 7.4. Growth Factors

Vascular endothelial growth factor (VEGF) is a signalling protein concerned with reversing inadequate blood circulation, which may arise from hypoxia linked to mechanical overload, through the stimulation of angiogenesis. VEGF recruits chondrocytes, osteoclasts, and endothelial cells during hypoxia [31,253]. Additionally, VEGF regulates MMPs and tissue inhibitors of metalloproteinases (TIMPs). The expression of VEGF in chondrocytes of the condyle was increased in correlation to the degree of applied mechanical stress in addition to the presence of osteoclasts in the tissue area where VEGF is highly expressed [254]. Cyclic tension upregulates the expression of VEGF and MMPs, while it downregulates the expression of TIMPs in chondrocytes [255]. Furthermore, VEGF upregulation in the synovial tissue and fluid is directly correlated to the degree of joint effusion in cases of internal joint derangement [253,256,257]. VGEF was also increased in human TMJ discs with varying degrees of disc tissue degeneration/regeneration [258], implying its role in TMJ inflammation.

Several other growth factors were detected in TMDs, including BDNF, fibroblast growth factor (FGF)-4, FGF-9, and insulin-like growth factor-binding protein-2, in correlation to joint effusion [256]. Together, VEGF and other growth factors activate chondrocytes to release MMPs and reduce TIMPs, resulting in an imbalance in the regulation of the extracellular matrix, leading to cartilage destruction accompanied by bone resorption due to osteoclast recruitment [259].

### 7.5. Proteoglycans

Proteoglycans, like aggrecan, fibromodulin, and biglycan, have an essential role in subchondral bone turnover in TMJ internal derangement. The deficiency of biglycan and fibromodulin in a mouse model resulted in the upregulation of the ratio between the receptor activator of nuclear factor kappa B and that of osteoprotegerin [260]. This leads to an increased bone turnover due to the regulation of osteoclasts by osteoprotegerin, highlighting the critical role of proteoglycans in maintaining subchondral bone integrity and their possible role in the early stages of OA. A higher concentration of aggrecan was detected in the disc of patients with chronic closed lock as compared to the aggrecan levels in patients with painful clicking [261].

Although there are different diagnostic procedures for TMDs, some limitations are still remarkable. Due to the rapid changes in therapy response, biological markers could become a critical part of the diagnostic process. They might provide relevant information more rapidly, which contributes to understanding the mechanisms underlying TMDs’ clinical efficacy.

## 8. Treatment Modalities of Temporomandibular Disorders

The traditional TMD treatments include physical therapies, occlusal splints, non-steroidal anti-inflammatory drugs (NSAIDs), muscle relaxants, low-level lasers, and arthrocentesis with lubrication or corticosteroid. A reduction in the muscle-related overload in patients with severe bruxism by splint utilization could be effective in inducing condylar bone remodelling [262] and improving the symptoms [263]. However, this treatment option appeared to be less efficient if not accompanied by counselling and masticatory muscle exercises [264]. Patients with TMD experience high levels of stress and anxiety. Adding psychological interventions such as cognitive behavioural therapy (CBT) to standard treatment regimens may improve patients’ quality of life, reduce muscle tension, and help patients cope with daily activities [265]. According to the findings of a systematic review, CBT and self-care management improve pain and disability in comparable ways; however, CBT is more effective at treating activity interference and depressive symptoms. CBT combined with standard treatment offers short-term improvements in pain and pain management compared to standard treatment alone [266].

A study conducted by Radwan et al. [267] demonstrated that the administration of oncologic dose of zoledronic acid in rats has a catabolic effect on the TMJ’s condyles after six weeks. However, after 12 weeks, this effect was reversed as evidenced by the observed enhanced endochondral and intramembranous ossification. Surgical intervention can be resorted to in severe cases in order to restore joint function. Surgical intervention includes joint replacement with an autologous bone or an artificial joint [268].

### 8.1. Surgical Treatment

Conservative treatment modalities for TMDs are usually the first line of treatment resorted to and are considered as the most effective approach in the majority of the patients. However, in severe, non-responsive cases, surgical procedures for TMD treatment may be regarded [269,270].

Surgical treatment options include minimally invasive, closed, TMJ procedures including arthrocentesis and arthroscopy in addition to open joint surgery, arthrotomy, including disc plication, disc repositioning, condylotomy eminectomy, and eminoplasty. Finally, if all other treatment modalities have failed, surgeon might resort to prosthetic total joint replacement [271,272,273,274]. The choice of surgical procedure depends largely on the patient’s condition in addition to the experience of the surgeon [270].

#### 8.1.1. Closed Temporomandibular Joint Procedures

##### Temporomandibular Joint Arthrocentesis

Despite being categorized as a surgical procedure, arthrocentesis is a non-surgical procedure. Even though it is invasive, the risk of causing harm to the surrounding soft tissues and joint structures is very low. Arthrocentesis entails cost-effective and more available tools and is carried out under local anesthesia. Despite the lack of direct visual inspection of the joint structures, it has gained a lot of popularity [275].

TMJ arthrocentesis comprises a small needle (19 to 21 gauge) used to puncture the upper joint cavity through the skin in front of the tragus for irrigation, and the fluid is then expelled through an outflow needle. Flushing out the pro-inflammatory cytokines and pain mediators are effective for reducing pain and disability [276]. It is indicated for localized joint pain and restricted joint movements like closed lock, anchored disc phenomenon, osteoarthritis, and different inflammatory diseases. It was demonstrated that the long-term outcomes of arthrocentesis range from 85% to 90%, regardless of the operator’s expertise [277].

##### Temporomandibular Joint Arthroscopy

Arthroscopy is a minimal invasive technique that relies on advanced technology and equipment [278]. It enables visualizing a three-dimensional space on a two-dimensional screen image through inserting a small arthroscopic telescope (1.8 to 2.6 mm wide) into the TMJ’s upper joint space and then connecting a camera to the telescope to display the image on a TV screen. Another access tool is positioned about 10 to 15 mm ahead of the arthroscopy that allows irrigation outflow and the insertion of instruments into the joint space [279].

This technique helps to assess the upper joint by identifying the tissue attachment at the posterior end, to examine the synovial lining for signs of inflammation, to detect clicking or limited movement of the disc joint, to identify the degenerative changes like softness, fibrillation or tears in the articular cartilage, to observe the eminence while moving the arthroscopy through the joint space, to asses any adhesions or other pathology in the joint space, and to identify the disc’s integrity and the tissue’s posterior perforations or attachment. Arthroscopy can be utilized in breaking up adhesions by moving either the arthroscopy or the irrigation cannula through the adhesions and separating them. Finally, it can aid in injecting steroids into the joint space or inflamed tissues directly [280]. The 1.2 mm arthroscopic telescope is an advanced improvement in arthroscopy that enables arthroscopic lysis and lavage procedures in the office under either local anesthesia or conscious intravenous sedation while providing the superior visualization of the joint structures. The initial findings of office-based arthroscopy showed equivalent outcomes to arthrocentesis and hospital-based arthroscopy for patients experiencing pain and limited mouth opening [277].

### 8.2. Open Joint Surgery

#### 8.2.1. Disc Repositioning

Cases of TMJ disc displacement are usually managed non-surgically or via arthrocentesis or arthroscopy, and cases that fail to respond well are usually managed through arthrotomy-based disc repositioning, discopexy, or even discectomy in severe cases. Studies have reported a good success rate for discopexy [281,282,283]. Arthrotomy-based discopexy aims to restore the normal anatomical position of an anteriorly displaced disc [284]. It is usually performed via endaural or pre-auricular incisions. It involves the anterior release of lateral pterygoid muscle attachment to the disc that is associated with retrodiscal tissue excision, thus allowing posterior disc repositioning, which is then held in place via a suture or tack to fixate the disc reduction [274,285,286]. In sever non-responsive cases, if the disc shows significant deformation that hinders the joint function and mobility, discectomy may be resorted to [287].

#### 8.2.2. Codylotomy

Condylotomy is an alternative for arthroplasty or interpositional arthroplasty for treating patients with TMDs, particularly in patients suffering from osteoarthrosis and internal joint derangement [288,289]. It aims to improve or normalize disc position by increasing the joint space, thereby alleviating symptoms like pain and mechanical issues [288]. This method avoids intracapsular changes that could occur with other surgical techniques. Even when used unilaterally, its application improves both joints [289]. To encourage condylar sag, a specific procedure called modified condylotomy that involves the removal of a portion of the medial pterygoid muscle is used. This aids in moving the disc back into a more typical position [290]. Among the disadvantages of modified condylotomy, it is not advised for joints with persistent, permanent disc displacement that do not have active osteoarthritis. Bite changes are possible, particularly in patients who have dentures or poor tooth intercuspation. In rare cases, the proximal segment may move medially, which could harm the nerve [288].

##### Eminectomy and Eminplasty

One of the most popular surgical techniques for treating recurrent TMJ dislocation is eminectomy, which is referred by many surgeons as a “rescue procedure” [291]. The process aims to eliminate the articular eminence so that the condyle can freely move backwards. Eminectomy was effectively used to treat recurrent TMJ dislocations, either by itself or in conjunction with other procedures [292,293,294,295]. A variation of complete eminectomy, known as reduction eminoplasty, is partial eminectomy. This process reduces the eminence somewhat rather than eliminating it. Compared to eminectomy, eminoplasty reduces the risk of perforation into the middle cranial fossa and is a dependable option in the event of eminence pneumatization [293]. Eminectomy was found to be superior to eminoplasty in a study that compared both the procedures regarding the recurrence, operation time, and TMJ pain after the respective procedure [296]. Patients with TMDs including anchored disc syndrome, habitual dislocation, and internal derangement showed excellent outcomes after treatment using arthroscopic eminoplasty. TMJ pain recorded 18 months following the surgery was lower than the pain measured 6 months prior to surgery. Additionally, the mouth opening after surgery was larger than the mouth opening before surgery [293,297].

##### TMJ Total Joint Replacement

TMJ total joint replacement is a critical surgical procedure designed to address severe TMJ disorders that do not respond to more conservative treatments. This operation involves the complete substitution of the damaged joint with an artificial implant, aiming to reduce pain and restore functionality in patients suffering from conditions such as degenerative joint disease, trauma, or structural deformities. The indications for TMJ total joint replacement include the following: (1) the failure of joint development, irrespective of etiology; (2) the irretrievable loss of joint tissues due to factors such as necrosis or neoplasm; and (3) the advanced degeneration of joint tissues after the exhaustion of less invasive treatment modalities, including conservative management, intra-articular injections, and arthroscopic techniques [298,299,300,301,302].

The replacement of the TMJ is a complex surgical procedure with a considerable risk of failure. Possible intraoperative outcomes include exposure to the cerebral cavity, impairment of motor and sensory innervation, and trauma to blood vessels and the parotid duct. Postoperative problems may include infection, implant rejection, cerebrospinal fluid leaking, salivary fistula, and facial deformity accompanied by unsightly scarring. Given the dangers mentioned above, temporomandibular joint prosthesis implantation is appropriate for instances where less invasive therapy modalities have proven ineffective [303,304,305,306].

The previous treatment modality does not fully restore the biological functions of the TMJ with long-term prognosis certainty. Most of the current treatments can effectively reduce pain, without a guaranteed therapeutic effect on the histopathological structure of the joint. Therefore, there is a need of treatment modalities that target the destructive biological markers or utilize biological supplements for deficiencies associated with TMD pathogenesis.

### 8.3. Cytokine-Based Therapy

The intra-articular injection of cytokines or anti-cytokines could be efficient in stimulating the repair of the joint’s cartilage. The intra-articular injection of IL-1ra or TNF-α inhibitors displayed promising results in the protection of knee joint cartilage [307]. Moreover, TGF-β1 efficiently promoted the synthesis of extracellular matrix in fibrochondrocyte and chondrocyte co-cultures [308] and efficiently increased the expression of proteoglycans in degraded cartilage, with a protective effect on the subchondral bone in a TMJ-OA in vivo model [309].

### 8.4. Non-Steroidal Anti-Inflammatory Drugs and Corticosteroids

The treatment potential of non-steroidal anti-inflammatory drugs (NSAIDs) could be referred mainly to cyclooxygenase (COX)-2 inhibition activity and the downregulation of cytokine-related injury. Within mandibular condylar chondrocytes exposed to increased cyclic tensile strain, it has been reported that the COX-2 inhibitor celecoxib inhibited the upregulation of COX-2, Prostaglandin E2 (PGE2), aggrecanase, and MMPs while upregulating type II collagen and aggrecan in vitro [310].

The intra-articular application of tenoxicam showed higher potency as compared to orally administered drugs regarding their anti-inflammatory and analgesic effects. Owing to the role of corticosteroids in the inhibition of the release of arachidonic acid, the source for prostaglandins and leukotrienes, intra-articular injections of glucocorticoids diluted with a local anesthetic solution are widely used in patients with TMDs. This method of administration is safe, and it guarantees a lower systemic exposure to corticosteroids with fewer side effects [311].

### 8.5. Autologous Conditioned Serum

Autologous conditioned serum (ACS), as a potent source of IL-1ra, effectively promoted knee joint cartilage and subchondral bone regeneration [312]. The effect of ACS on TMDs warrants evaluation, as IL-1β is a key player in the initiation and progression of the disease.

### 8.6. Hyaluronic Acid

Hyaluronic acid (HA) can be normally detected in the synovial fluid and TMJ cartilage matrix. The intra-articular injection of HA can be used for the management of variable TMDs [313,314]. Moreover, the intra-articular injection of HA was found to be superior compared to NSAIDs [315] and with similar effectiveness when compared to corticosteroids [311] in relieving the symptoms of TMJ-OA. Positive TMD treatment results were attained upon the injection of HA into the upper or lower joint space; however, the injection of HA into the lower joint space resulted in better remodelling of the condyle [316]. A systematic review revealed the beneficial effects of HA on the regulation of inflammatory mediators, including nitric oxide and plasminogen activator system, associated with TMJ-OA in humans [317].

### 8.7. Glucosamine

Glucosamine is the metabolic precursor of aggrecans and other proteoglycans in the cartilage. Glucosamine could inhibit cartilage degeneration and promote proteoglycan synthesis. Oral glucosamine is prescribed as a safe, over-the-counter adjunct to HA injection in treating TMJ-OA [318].

### 8.8. Bioactive Compounds

Naturally occurring bioactive compounds with anti-inflammatory and analgesic effects have potential applications in the development of medications for TMD treatment [280]. However, studies exploring the ideal dosage and possible side effects of these natural compounds are still deficient. Therefore, preclinical research on these compounds should be emphasized.

Lectins are a heterogeneous group of proteins that can specifically and reversibly bind to simple sugars or complex carbohydrates. Lectins extracted from various plants showed promising results in treating TMDs. Lectin has anti-inflammatory [319,320,321] and anti-nociceptive actions [321,322] in TMD induced models. Moreover, moringa oleifera Lam is a plant that can be found in the tropics. A set of derivatives of extractions from Moringa oleifera, MC-D7, MC-D9, and MC-Hl, were reported to be safe and efficient analogues in reducing the TMJ hypernociception, along with the diminished plasma extravasation, showing anti-inflammatory activity in formalin-induced TMJ hypernociception in a rat model [323]. Additionally, Tephrosia toxicaria Pers is a plant known for its pain and inflammation relief. These effects were observed in mice through the inhibition of pro-inflammatory cytokines, such as TNF-α and IL-1β, along with the NO-dependent leukocyte recruitment inhibition [323].

Elevated levels of oxidative stress biomarkers, along with reduced antioxidant capacity, were observed in patients with TMDs [210]. The Euphorbia bicolor Latex extract significantly reduced pro-inflammatory cytokines/chemokines and oxidative stress biomarkers in a rat model of orofacial pain and was associated with a significant reduction in pain [324]. Terpene is a structurally diverse large category of molecules that include both primary and secondary metabolites. α-bisabolol (BISA), a class of terpene, was confirmed as an antagonist to transient receptor potential ankyrin 1 (TRPA1) and is associated with TNF-α reduction and is effective in treating TMD-associated pain [325]. Furthermore, resveratrol is a natural bioactive compound extracted from multiple plants, like grapes. It demonstrated potent anti-inflammatory properties, and it was successfully used to treat complete Freund’s adjuvant-induced inflammatory TMD models [326].

### 8.9. Delivery System for Therapeutic Agents in TMD Treatment

#### 8.9.1. Orally Delivered Agents

##### Estrogen

The estrogen hormone was associated with the retardation of fibrocartilage degenerative changes by upregulating the anabolic genes in the articular fibrocartilage. Additionally, estradiol via the estrogen receptor alpha successfully decreased protease gene expression and cleavage of collagen (Col)1 and Col2 in the fibrocartilage matrix [327,328]. However, the estrogen’s effect on TMDs in humans showed a divergent and occasionally contradictory effect [329]. A review recommended that sex- and age-related estrogen signalling matters when evaluating the effect of estrogen in TMDs, thus specific drugs should be industrialized [330].

##### Vitamin B Complex

Vitamin B is a water-soluble vitamin that can be used as an analgesic drug and as a promoter of the analgesic effect of diclofenac for reducing treatment duration [331,332]. Vitamin B revealed stronger analgesic property compared to vitamin E and diclofenac in treating patients with knee OA [333]. This anti-inflammatory and analgesic effect is related to the cyclooxygenase pathway and opioid receptors [334]. Vitamin B can be a valuable adjuvant in treating TMDs.

#### 8.9.2. Intra-Articular-Injection- or Intramuscular-Injection-Delivered Agents

##### Platelet-Rich Plasma (PRP)

PRP is rich in variable growth factors, which significantly improve bone formation in surgically induced degenerative changes in the TMJ of rabbits but showed no marked effect on cartilage degradation repair [335]. PRP demonstrated superior therapeutic potential in treating TMJ-OA, showing greater long-term improvements in pain reduction and mouth opening compared to HA therapy [336,337] and Botox [18]. A number of randomized clinical trials and observational studies revealed the effectiveness of PRP in alleviating the pain symptoms associated with TMDs [337,338,339,340,341,342,343].

##### Ozone

For medical purposes, the potent oxidant, ozone gas, is administered as ozonized oil besides its gaseous state. The intra-articular administration of ozone gas has demonstrated positive outcomes by enhancing the joint-repairing capabilities of fibroblasts and exhibiting strong anti-inflammatory and chondrogenic effects, making it a promising treatment for the internal derangement of the TMJ [344].

##### Botulinum Toxin

Over the past 20 years, botulinum toxin (BTX) has been used to treat head and neck pain [345]. It is a 150 kDa exotoxin produced by Clostridium botulinum, and seven serotypes exist, with serotype A (BTX-A) being the most available type. BTX possess analgesic effects and can successfully reduce the parafunctional motions involving the masticatory muscles, which account for its potential role in TMD therapy [346].

In a randomized controlled clinical trial, botulinum toxin type-A (BTX-A) and low-level laser therapy (LLLT) were found effective in managing patients with symptomatic TMJ disc displacement with reduction. BTX-A and LLLT caused a rapid relief of symptoms with a significant reduction in pain and a change in the disc–condyle relationship [347].

Additionally, Botox injection and dry needling showed promising results in the management of pain and in the restoration of functions in patients with myofascial pain syndrome in the TMJ [348,349].

#### 8.9.3. Transdermally Delivered Agent

Cannabis sativa-related drugs may hold promise in pain relief in TMDs. Cannabidiol (CBD) cream significantly counteracted the encephalomyelitis (EAE)-related neuroinflammation and neurodegeneration through reducing the release of CD4 and CD8 T cells, in addition to reducing the expression of the main pro-inflammatory cytokines, oxidative injury, and apoptosis [350]. Furthermore, the transdermal delivery of the CBD compound showed positive results in treating painful peripheral neuropathy and TMDs, providing positive evidence supporting the use of CBD for the management of neuroinflammation and neurodegeneration [351].

### 8.10. Regenerative Medicine

Regenerative medicine emphasizes the utilization of stem/progenitor cells alone or loaded on scaffolds to construct or restore body tissues. Owing to their relative accessibility and differentiation ability, mesenchymal stem cell (MSC) therapy is a promising candidate in TMD regenerative therapy. Resident MSCs have been isolated from the synovia of the TMJ, suggesting their important role in TMJ repair [352]. Upon culturing MSCs in osteogenic and chondrogenic media for 7 days and seeding them on hyper-hydrated collagen gel, bone- and cartilage-like tissue formations were observed [353]. Previous reports indicate that MSCs injected into the upper compartment of the TMJ can survive for up to four weeks, as evidenced by in vivo tracing [354]. These MSCs show promising effects on the restoration of joint cartilage, especially following in vitro chondrogenic differentiation [355]. Human umbilical cord matrix mesenchymal stem cells (hUCM-MSCs) showed a significant anti-inflammatory effect in induced TMJ-OA in rabbit model, which was comparable to that of corticosteroids. Their potent effect was attributed to the upregulation of growth factors, extracellular matrix markers, and anti-inflammatory cytokines including TGF-β1 and IL-10 expression, besides the downregulation of the expression of pro-inflammatory cytokines including TNF-α, IL-1β, IL-6, and IL-17 [356]. The intra-articular injection of microvesicles in osteoporotic rats revealed a marked improvement in bone architecture, significant increase in osteogenic markers (alkaline phosphatase, bone morphogenetic proteins, and Runt-related transcription factor 2), and a significant decrease in inflammatory markers (IL-1β, TNF-α, and IL-6), as well as in the receptor activator of nuclear factor kappa beta ligand expression [10].

Challenges for this approach include the optimal selection of cells, growth factors, and scaffold materials. Additionally, the utilization of other types of MSCs including oral or dental MSCs should be thoroughly explored for the management of TMDs.

## 9. Conclusions

The multifactorial pattern of TMDs dictates a personalized treatment starting from an overall evaluation of the case through accurate diagnosis and postural and gnathological examinations to correlate the disorders reported by the patient and their actual cause. The challenge is the removal of the etiology, stopping the disease progression, and treating the TMD in synchrony. This requires the initial supportive patient learning for TMD treatment. Adjunctive measures include a soft diet to rest the jaws, moist, warm wrappings, and stretching exercises. Moreover, cognitive behaviour therapy and biofeedback in pain management are necessary. Patients should be advised for behaviour modifications such as stress reduction, sleep hygiene, abolition of parafunctional movements like teeth grinding and clenching, and avoidance of risky mandibular movement.

Pro-inflammatory cytokines, including IL-1β, TNF-α, IL-6, and IL-8, mediate osteoclast differentiation, leading to bone resorption and metabolic imbalance in TMJ chondrocytes. These processes contribute to the etiopathology of TMJ osteoporosis and osteoarthritis. Therefore, being common in TMJ disorders, these biological markers could be used for proper diagnosis since the clinical picture of some TMJ pathologies might be unknown. Recent treatment modalities targeting pro-inflammatory cytokines could be mandatory and need to be translated clinically.

Efforts should be directed toward regenerative medicine and tissue engineering in treating TMDs. This up-to-date modality allows the repair and regeneration of the TMJ cartilage and subchondral bone, providing long-term solutions. Employing MSCs, microvesicles, and PRP rich in growth factors are promising in terms of downregulating pro-inflammatory cytokines along with enhancing osteoblastogenesis. More studies are needed to analyze the involvement of the different biological markers in TMDs with evidence of its role in disease progression, which enables the development of a pharmaceutical agent for therapy, such as targeting TIMPs to counteract the effect of MMPs.
